# A Case Report of a Rapid Development of Hepatocellular Carcinoma (HCC) Within Six Months of Hepatitis C Cure in an Individual With Risk Factors

**DOI:** 10.7759/cureus.79571

**Published:** 2025-02-24

**Authors:** Muhammad A Israr

**Affiliations:** 1 Internal Medicine, Fairfield Medical Center, Lancaster, USA

**Keywords:** alcoholic cirrhosis, gastrointestinal oncology, hepatitis c (hcv) infection, hepatocellular carcinoma (hcc), injection drug use

## Abstract

People with chronic liver disease are more likely to develop hepatocellular carcinoma (HCC), especially those with cirrhosis or fibrosis. Confounding variables, such as alcohol consumption, end-stage renal disease, and poorly controlled diabetes mellitus, can lead to detrimental outcomes such as the development of HCC since the liver is already damaged and in a recovery phase from the resolved hepatitis C infection. HCC should be a high differential diagnosis even in the absence of classical signs and symptoms of jaundice or weight loss given the resolved hepatitis C infection. In this case study, a 63-year-old male with a past medical history of intravenous (IV) drug use, chronic alcoholic cirrhosis, end-stage renal disease, and cured hepatitis C infection presented at the primary care office for a regular follow-up visit after getting discharged from the emergency department (ED). During a routine primary care visit, the patient complained of right upper quadrant pain, constipation, and intermittent dizziness. At the time, he also endorsed drinking a case of beer daily and a fifth of liquor monthly. He had a history of hepatitis C, which he acquired through IV drug use. He was successfully treated with a six-month course of glecaprevir/pibrentasvir with eradication of the virus. Before the primary care practitioner (PCP) visit, the patient had an ED visit for abdominal pain and chronic constipation, during which he underwent a non-contrast CT of the abdomen, with an incidental finding of a 2.4 cm liver mass in the right hepatic lobe. It was followed up with an MRI and CT-guided biopsy, the results of which showed poorly differentiated HCC.

## Introduction

Hepatocellular carcinoma (HCC) is one of the leading causes of cancer-related deaths in the US, with more than 80% of cases occurring in patients with cirrhosis. The most common risk factors for HCC, in addition to male sex and older age, include hepatitis B virus (HBV) infection, hepatitis C virus (HCV) infection, metabolic associated steatotic liver disease (MASLD), alcohol consumption, diabetes mellitus, and smoking. According to the American College of Gastroenterology, it is estimated that HCC develops in about 2%-3% of US patients with cirrhosis, regardless of the cause [1]. As cirrhosis etiologies shift, HCC risk is shifting from predominantly viral (HCV, HBV) to eradicated HCV and nonviral (MASLD and alcohol-related liver disease) causes, with lower incidence rates. In this case, despite being cured of the HCV infection, a 63-year-old male ended up developing HCC in a relatively fast manner; therefore, his risk factors, such as diabetes mellitus, end-stage renal disease, and daily alcohol use, played a significant role in the development of HCC. The mass was detected incidentally on a CT of the abdomen during an ER visit for abdominal pain and constipation. Follow-up studies with MRI and CT-guided biopsy revealed poorly differentiated HCC.

## Case presentation

**Figure 1:**
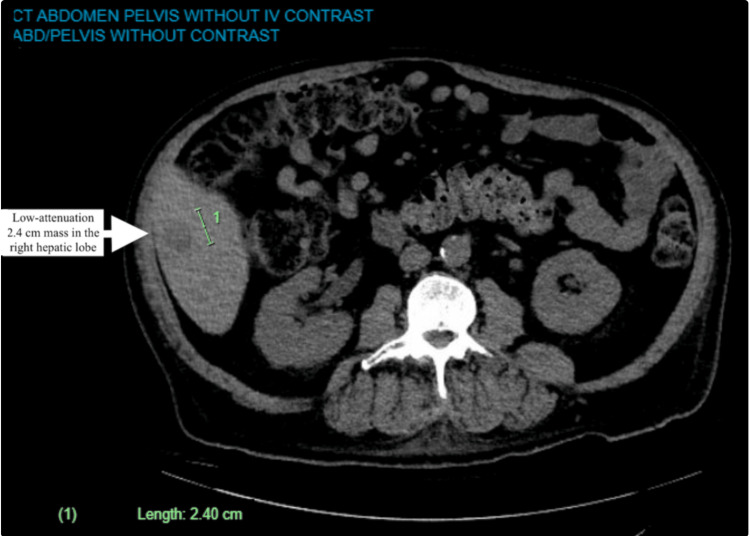
CT scan of the abdomen, with the white arrow showing the initial mass size

The patient is a 63-year-old male with a past medical history of uncontrolled type 2 diabetes mellitus, intravenous (IV) drug use, alcoholic cirrhosis, end-stage renal disease, cured hepatitis C infection, status post resection of ampullary adenoma, cholecystectomy, chronic alcohol use, and chronic smoker, who continues to drink alcohol and smoke cigarettes initially presented three months after being cured for HCV to an emergency department with complains of non-specific abdominal pain and constipation. The patient had a chronic hepatitis C infection diagnosed two years before this presentation in the ED. During the initial lab work, an HCV polymerase chain reaction (PCR) showed an increased viral load of 2,388,410 IU/mL, which after the treatment was undetectable. During the workup, a CT of the abdomen/pelvis in the ED showed a new low-attenuation 2.4 cm mass in the right hepatic lobe (Figure 1).

He was discharged and instructed to follow up with a primary care practitioner (PCP). Upon presentation at the PCP's office, his abdominal pain had resolved, and he continued to have occasional constipation. In addition, he endorsed continued drinking alcohol, roughly a fifth monthly. PCP ordered a follow-up MRI scan of the abdomen, AFP levels, and a specialist referral. Alpha-fetoprotein (AFP) before treatment was 63.5 ng/mL, and at the time of HCC diagnosis, it was elevated at 15,686 ng/mL.

**Figure 2:**
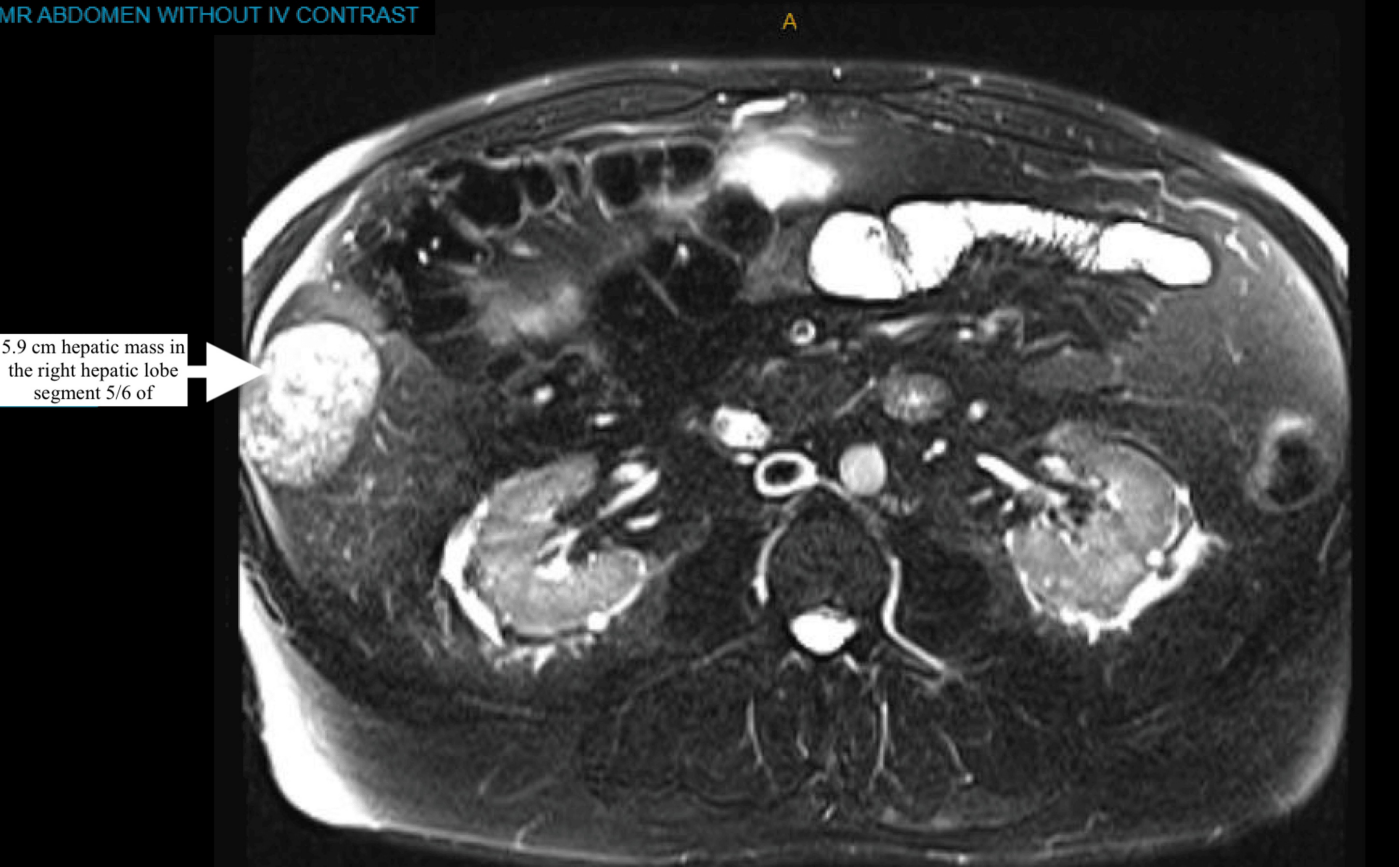
MRI of the abdomen, with a white arrow showing an increase in mass size

Further imaging with an MRI of the abdomen showed a rapidly increasing size to a 5.9 cm hepatic mass in the right hepatic lobe segment 5/6 of the liver, highly suspicious for malignancy (Figure 2). A CT-guided biopsy of the liver mass with histological evaluation revealed poorly differentiated HCC. The patient denied symptoms of weight loss, jaundice, satiety, pruritus, and fatigue at the time of primary care evaluation. Physical exam findings were unremarkable for jaundice, except that he appeared to have an enlarged liver margin and mild distention with no signs of acute abdomen.

## Discussion

It can be inferred that consuming more than 80 g of alcohol per day elevates the risk of HCC by five times. Meanwhile, having HCV by itself heightens the risk of HCC by 20 times. It is estimated that the chance of developing HCC increases by more than 100-fold when both risk factors are present simultaneously [2]. There are several pathways involved in the development of HCC associated with HCV: fibrosis caused by continual necrosis; immune-surveillance failures caused by continuous viral replication, leading to immune system escape with direct carcinogenic proteins of HCV proteins; and immune system failures causing DNA mutations in liver cells by deregulating host cell cycle checkpoints. No clear explanation exists for how HCC develops after HCV cure [3]. One hundred eighty-six genes were expressed with a co-expression signature in chronic hepatitis C (CHC) patients with HCC, suggesting a virus-induced transcriptional reprogramming [4]. Changing epigenetic factors, such as histones, can result in chromatin opening and compacting that affect gene regulation. Hamdane et al. examined the effects of HCV-induced epigenetic alterations on HCC risk in humanized mice and patients. H3K27ac was found to be altered genome-wide following chronic HCV infection. A correlation was found between 5,318 modified genes and changes in mRNA and protein expression associated with CHC [5]. HCV cure with direct-acting antivirals (DAA) resulted in altered pathways due to epigenetic changes. Several factors are involved, some of which include tumor necrosis factor signaling inflammation, the G2M checkpoint, epithelial-mesenchymal transition, phosphoinositide 3-kinase, Akt, and the mammalian target of Rapamycin. After the cure for those with stage F2-3 fibrosis, H3K27ac changes observed in HCV-infected patients were partially reversed. H3K27ac changes persisted in 96.6% of DAA-cured patients with cirrhosis (stage F4).

Additionally, Perez et al. demonstrated that HCV infection induces genome-wide epigenetic changes through chromatin immunoprecipitation following next-generation sequencing of histone post-translational modifications [6]. In a study by Santangelo et al., exosomal microRNAs (miRs) were examined in CHC patients for innate immune response modulation by DAAs. It has been shown that miR-122 plays a role in HCV replication, and its loss is associated with the development of HCC. The study demonstrated that miR-122-5p, miR-222-3p, miR146-5p, miR150-5p, miR-30C-5p, miR-378a-3p, and miR-20a5p were enriched in exosomes derived from HCV-infected cells [7]. VEGF is a cytokine that affects cancer cell growth and survival in human HCC cells. A correlation exists between liver cancer angiogenesis and proliferative activity and VEGF expression. In a study, Villani et al. examined the effect of VEGF induced by DAA treatments on the angiogenesis of HCC. A fourfold increase in VEGF was observed in a study consisting of 117 cirrhotic patients treated with DAA [8]. Although VEGF levels decreased to normal 12 weeks after DAA treatment, the carcinogenesis remained, which could indicate an accelerated proliferation of cancer cells before HCV cure. The American Association for the Study of Liver Diseases (AASLD) recommends the surveillance of adults with cirrhosis because it improves overall survival. The AASLD recommends ultrasound surveillance, with or without AFP, every six months. AASLD recommends not performing surveillance of patients with cirrhosis with Child's class C unless they are on the transplant waiting list, given the low anticipated survival for patients with Child's class C cirrhosis [9]. As shown in Table 1 and Table 2, Marrero et al. demonstrated in the Journal of Hepatology a structured approach to identifying and managing HCC based on imaging findings for surveillance or intervention [10].

**Table 1:** Surveillance table Source: Ref [10]

Category	Details
Initial Test	Surveillance ultrasound (US) with or without alpha-fetoprotein (AFP).
Interpretation	Negative: Repeat ultrasound with or without AFP in 6 months.
	Subthreshold: Lesions <10 mm require repeat ultrasound in 3–6 months.
	Positive: Lesions ≥10 mm or AFP >20 ng/mL prompt multiphase CT or MRI.
Follow-up Imaging	In select patients, multiphase CT or MRI is performed.

**Table 2:** Diagnosis table Source: Ref [10]

Category	Details
Diagnostic Imaging	Multiphase CT or MRI is used for HCC evaluation
Interpretation	No observation detected: Return to surveillance in 6 months.
	No observation detected: Return to surveillance in 6 months.
	LI-RADS NC (Noncategorizable): Repeat or alternative diagnostic imaging in ≤3 months.
	LI-RADS 1 (Definitely Benign): Return to surveillance in 6 months.
	LI-RADS 2 (Probably Benign): Return to surveillance in 6 months; consider repeat imaging in ≤6 months.
	LI-RADS 3 (Intermediate): Repeat or alternative diagnostic imaging in 3–6 months.
	LI-RADS 4 (Probably HCC): Multidisciplinary discussion for further evaluation, including biopsy or repeat/alternative diagnostic imaging in ≤3 months.
	LI-RADS 5 (Definitely HCC): HCC confirmed.
	LI-RADS M (Malignant, not definitely HCC): Multidisciplinary discussion for tailored workup, biopsy (in most cases), or repeat/alternative imaging in ≤3 months.
Biopsy	In cases where a biopsy is performed, the diagnosis is confirmed pathologically.

## Conclusions

HCC remains a significant concern in the DAA era, especially among people with advanced hepatic fibrosis. The development of HCC predictive models is promising, but most still need validation and standardization. There is still a lack of understanding of the pathogenesis of HCC after HCV cure. Even after successfully treating HCV infection, factors such as diabetes mellitus, end-stage renal disease, and ongoing alcohol use are associated with the accelerated progression of HCC. These variables play a significant role in persistent damage to the liver. It may be possible to identify novel biomarkers to detect HCC early with the help of an understanding of the molecular mechanisms leading to the disease. Hence, regular monitoring with ultrasounds and tumor markers is essential for tracking HCC development and progression in high-risk patients, as it is both life-saving and cost-effective.
